# A Study on the Effect of Conductive Particles on the Performance of Road-Suitable Barium Titanate/Polyvinylidene Fluoride Composite Materials

**DOI:** 10.3390/ma18051185

**Published:** 2025-03-06

**Authors:** Zhenhua Zhao, Rui Li, Chen Zhao, Jianzhong Pei

**Affiliations:** 1 China Municipal Engineering, Northwest Design & Research Institute Co., Ltd., No.459 Dingxi Road, Lanzhou 730000, China; z17749028768@126.com; 2 Changan University, School of Highway, 126 Middle Sect, South Second Ring Rd, Xi’an 710064, China; lirui@chd.edu.cn (R.L.); peijianzhong@126.com (J.P.)

**Keywords:** barium titanate, polyvinylidene fluoride, conductive carbon black, graphene, piezoelectric properties, mechanical properties

## Abstract

The design of piezoelectric roads is one of the future directions of smart roads. In order to ensure the environmentally friendly and long-lasting use of piezoelectric road materials, lead-free piezoelectric ceramics (barium titanate), polymer piezoelectric materials (polyvinylidene fluoride), and conductive particles (conductive carbon black and graphene) were used to prepare composite piezoelectric materials. The electrical performance was studied by the conductivity, dielectric properties, and piezoelectric properties of the composite materials. Then, the mechanical properties of the composite material were investigated by load compression tests. Finally, the microstructure of the composite materials was studied. The results showed that as the amount of conductive particles increased, the electrical performance was improved. However, further addition of conductive particles led to a decline in the electrical performance. The addition of conductive particles had a minimal effect on the mechanical properties of composite materials. The composite material met road use requirements. The overall structure of the composite materials was compact, with a clear wrapping effect of the polymer, and good interface compatibility. The addition of conductive carbon black and graphene had no significant impact on the structure of the composite materials.

## 1. Introduction

Roads are vast energy fields. The frequent impact loads caused by vehicles driving on the road cause vibrations and deformations, which ultimately result in mechanical energy loss and significant road damage [1]. Therefore, harvesting the mechanical energy will not only increase the service life of the road but also provide a broader perspective for the future of the road. Piezoelectric materials are intelligent functional materials that convert mechanical energy into electrical energy. The miniature power generation devices are embedded beneath the road surface, which generate electricity under vehicle loads. Given the large road network in China, and the high car ownership, the construction of power-generating roads holds promising prospects. To collect this mechanical energy, developing a piezoelectric material suitable for road applications is of paramount importance, and energy harvesting needs to be resolved urgently. Although roads possess a certain load-bearing capacity during service, some deformation and damage are inevitable. Therefore, piezoelectric materials used on roads must have mechanical strength and the ability to withstand a certain amount of deformation recovery. However, most piezoelectric oscillators or transducers are made of pure ceramic components, and the brittleness of ceramics severely limits their application and lifespan. Research on piezoelectric materials mainly focuses on lead zirconate titanate (PZT) ceramics, which have high piezoelectric properties. In the 1950s, PZT emerged as a new type of piezoelectric ceramic and became widely applied, capturing 90% of the market share. Researchers subsequently developed binary, ternary, and even quaternary PZT piezoelectric ceramics, which were widely used in sensors, vibration meters, flow meters, ultrasound detectors, concrete inspection devices, and other electronic components. Especially in low-frequency and high-power piezoelectric devices, piezoelectric ceramics play an irreplaceable role. Although PZT ceramics possess high piezoelectric performance, their high lead content (about 70%) severely limits their usage in today’s society, especially in road applications. Since road materials are in direct contact with the ground, and road material recycling is difficult, this leads to significant lead infiltration into the land, potentially threatening the natural environment and even causing severe environmental pollution. As a result, further research and application of PZT ceramics for road use are no longer valuable. To apply piezoelectric materials on roads, lead-free and environmentally friendly materials are a must. Given these challenges, the development of road self-power generation technology by converting road vibration energy into electrical energy, from a materials science perspective, represents the future direction for road piezoelectric functional materials.

Barium titanate (BaTiO_3_) ceramics are widely used in electronic, mechanical, and other components. However, due to their relatively low piezoelectric properties and the lack of effective methods to significantly improve their performance at the time, BaTiO_3_ ceramics did not see much progress in some important fields. However, as research on PZT ceramics deepened and ideas broadened, researchers began to revisit BaTiO_3_. Studies have shown that the grain size significantly affects the piezoelectric performance of BaTiO_3_ ceramics. Japanese scholars Takahashi et al. [2] used a hydrothermal method to produce nano-scale BaTiO_3_ ceramics with small particle sizes, achieving a piezoelectric strain constant (d_33_) of up to 350 pC/N. Zheng et al. [3] prepared BaTiO_3_ powder with a particle size of 0.94 μm, achieving a d_33_ value of 338 pC/N. In 2008, Takahashi et al. [4] used microwave sintering to sinter nano-scale ceramics, producing BaTiO_3_ ceramics with a d_33_ value of 350 pC/N. Karaki et al. [5] used a two-step sintering method to obtain BaTiO3 ceramics with a d33 value of 460 pC/N. Huan et al. [6,7] used a two-step sintering technique on 1 μm BaTiO3 powder, with the final piezoelectric ceramic sample achieving a d_33_ value of 519 pC/N, surpassing general PZT ceramics. According to statistical data, the d_33_ value of general PZT piezoelectric ceramics ranged from 260 to 600 pC/N. Liu et al. [8] introduced a lead-free piezoelectric material, Ba(Ti_0.8_Zr_0.2_)O_3_−(Ba_0.7_Ca_0.3_)TiO_3_, with a d_33_ value of up to 620 pC/N, which completely exceeded the maximum d_33_ value (600 pC/N) of PZT ceramics. While research on BaTiO_3_ ceramics had stagnated due to their inherent properties, recent studies have shown that proper treatments could significantly enhance the piezoelectric properties of BaTiO_3_ ceramics. Due to its low cost, higher piezoelectric performance, and inherent lead-free and environmentally friendly properties, BaTiO_3_ is undoubtedly a preferred choice for road piezoelectric materials, and further research on its potential must be reconsidered.

Despite the excellent piezoelectric performance of piezoelectric ceramics, they have many drawbacks for road applications. Given their inherent brittleness and tendency to fracture, piezoelectric oscillators or transducers made of ceramics do not possess good mechanical tensile toughness or seismic resistance. If they are embedded in road structures, they are likely to break under the impact load of vehicles, and long-term driving will cause damage to the piezoelectric oscillators or transducers. This not only damages the road structure but also makes recycling and replacement difficult, posing long-term environmental risks. The first piezoelectric composite material came out in Japan. In 1972, Japanese scholar Nakayama developed the BaTiO_3_/PVDF composite by combining BaTiO_3_ ceramics and PVDF polymers. This breakthrough was significant in the development of piezoelectric composites. Han et al. [9] improved the properties of PLZT/PVDF by adjusting the ratio of La elements in PZT ceramics, which enhanced the overall piezoelectric and dielectric properties of the composite, raising the d33 value from 53 pC/N to 76 pC/N and the εr value from 159 to 222, with a 40% increase in both properties. At the same time, the influence of ceramic-phase particle size on the performance of the composite material was considered. Li et al. [10] also studied the effect of ceramic-phase particle size, showing that the piezoelectric properties of the composite reached their peak when the PZT particle size was between 43 and 75 μm. Guan et al. [11] incorporated carbon nanotube conductive particles into PZT/PVDF composites, enhancing both piezoelectric and dielectric properties. Zhang et al. [12] mixed nano-iron with BaTiO_3_ ceramics and added them as fillers to PVDF polymers. Their experiments showed that the composite’s relative dielectric constant reached 385 when the amount was 33%. Li et al. [13] incorporated graphene flakes into BaTiO_3_/PVDF composites, improving the dielectric constant while reducing dielectric loss.

This paper addressed the practical application of power-generating roads, faced with the reality that piezoelectric ceramics have excellent piezoelectric properties but are brittle. Polymers, although having poor piezoelectric properties, are easy to thermally press and have superior mechanical properties. This study combined the excellent piezoelectric properties of ceramics with the excellent mechanical properties of polymers to prepare road-suitable piezoelectric composites. From an environmental protection perspective, lead-free barium titanate (BaTiO_3_) and polyvinylidene fluoride (PVDF) were chosen to prepare the composites. The effects of adding conductive carbon black and graphene on the electrical performance of lead-free piezoelectric composites were investigated, and the mechanisms were explored. The optimal contents amount of conductive particles was determined, and the mechanical properties of the composites were tested to study the impact of conductive particles on the mechanical performance. Finally, the microstructure of the composite materials was studied. The goal was to develop high-performance, road-suitable, lead-free piezoelectric composites.

## 2. Materials and Methods

### 2.1. Materials

The BaTiO_3_/PVDF piezoelectric composite material selected in this study consists of piezoelectric ceramics (barium titanate, BaTiO_3_) as the filler, uniformly dispersed in the polymer (polyvinylidene fluoride, PVDF), and then formed by hot pressing. The BaTiO_3_ powder is manufactured by Shandong Guoci Functional Materials Co., Ltd. (Shandong, China). The PVDF is a powder material produced by Shanghai San-Afu New Materials Co., Ltd. (Shanghai, China), product code FR901. The non-sintering conductive silver paste is a fluid material produced by Yute New Materials Co., Ltd. (Guangzhou, China) product code TYD-110Y, with a curing point of 110 °C. BP200 conductive carbon black from Cabot Corporation (Boston, USA) and m5-type few-layer graphene from Fuzhou Yihuan Carbon Co., Ltd. (Fuzhou, China) were used.

### 2.2. Preparation Method

#### 2.2.1. Preparation Method of BaTiO_3_

There are several methods for the industrial-scale production of barium titanate ceramics, including co-precipitation, solid-state, and hydrothermal methods. The solid-state method is reliable, cost-effective, and suitable for large-scale production. The co-precipitation method does not require high-temperature conditions and offers high product purity and good reactivity. The hydrothermal method, while relatively simple and involving low reaction temperatures, also offers high purity, controlled particle size, and excellent crystalline properties, though the production cost is relatively higher.

**Figure 1:**
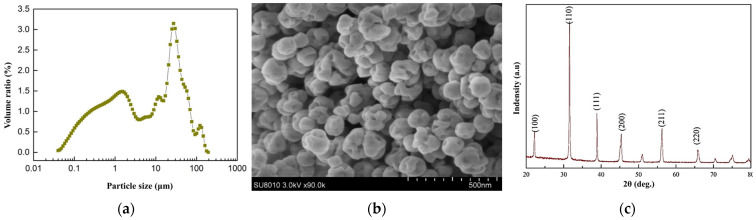
The particle size distribution, SEM, and XRD images of BaTiO3 made by hydrothermal synthesis: (**a**) particle size distribution; (**b**) SEM; (**c**) XRD.

The particle size distribution, SEM, and XRD images of BaTiO_3_ made by hydrothermal synthesis are shown in Figure 1. The particle size distribution of the BaTiO_3_ ceramic was relatively wide, the largest particle size did not exceed 200 μm, the smallest particle size was not less than 0.01 μm, and most of the particle size was concentrated in the range of 10~100 μm. However, from the SEM image, it could be seen that the BaTiO_3_ particles had a certain agglomeration phenomenon due to the action of surface energy, and the particles were more uniform in size after agglomeration. The shape of the BaTiO_3_ particles was irregularly spherical, and the difference in particle size was small. This represented that the size of the BaTiO_3_ had less effect on the properties as its size in the composite materials was the size of its agglomerated particles. Therefore, the BaTiO_3_ made by hydrothermal synthesis had a uniform size and distribution. The main diffraction peaks of BaTiO_3_ ceramics in the (110) crystal plane were found to be higher in peak value and intensity, and it can be seen from the figure that the diffraction peaks had lower baselines and sharper peak shapes, which suggested that the BaTiO_3_ crystals grew optimally along the (110) crystal plane. It was also found that there were stronger diffraction peaks in the (100), (111), and (200) crystal planes of the BaTiO_3_ crystals, which indicated that the BaTiO_3_‘s crystallinity was better and it was a purer tetragonal chalcogenide structure, and also confirmed that the BaTiO_3_ ceramics possess certain piezoelectric properties. The hydrothermal synthesis of BaTiO_3_ powder involved treating a barium hydroxide solution containing specific substances under hydrothermal conditions to obtain high crystallinity, purity, fine particle size, and uniformity of the powder material.

#### 2.2.2. Preparation Method of BaTiO_3_/PVDF Composite Materials

**Figure 2:**
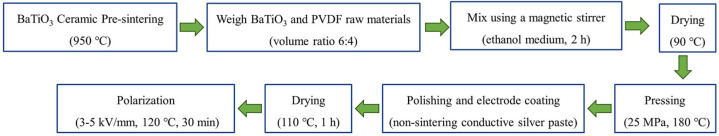
The preparation method of BaTiO_3_/PVDF composite materials.

The preparation process of the BaTiO_3_/PVDF composite materials is shown in Figure 2, which mainly involves three steps: hot pressing, electrode coating, and polarization. Hot pressing is a method that integrates heating and pressing for material formation. Compared to cold pressing, it offers unique high-temperature forming conditions. The materials selected for this experiment include BaTiO_3_ ceramic powder, which can only be sintered at temperatures above 1000 °C, and PVDF polymer, which liquefies at around 175 °C and solidifies rapidly below this temperature. The ceramic phase in the composite materials accounts for 60% of the volume.

During the hot-pressing process, the piezoelectric ceramic powder was thoroughly mixed with the polymer matrix. The polymer melted upon heating, allowing the ceramic powder to be encapsulated by the polymer. This resulted in the ceramics staying bonded together without undergoing sintering into a ceramic structure, while still maintaining certain mechanical strength. The composite materials reduced the brittleness of the ceramics while enhancing the overall mechanical toughness. The BaTiO_3_/PVDF powder, which had been evenly mixed and prepared, was weighed to a specific amount (sample size: 13 mm in diameter, 1 mm thick). For this experiment, 0.6 g of the powder was used. The hot-pressing mold temperature was set to 180 °C, and the pressing machine pressure was set to 25 MPa. Once the mold temperature reached 175 °C, the weighed powder was added to the mold. When the temperature reached 180 °C, the pressing machine was activated, and the timing began. Finally, when the hot-pressing time reached 30 min, the pressure was released from the pressing machine, the mold was closed, and the sample was removed after cooling to room temperature, completing one sample pressing process.

In the polarization process, polarization electrodes must be applied to both surfaces of the piezoelectric material, and a high voltage must be applied across these electrodes to complete the polarization. Since the piezoelectric composite used in this experiment contains polymer, the conventional surface silver paste infiltration could not be applied. Therefore, a no-bake TYD-110Y conductive silver paste was used instead. This conductive silver paste was evenly applied by hand to both electrode surfaces of the piezoelectric material and then dried at a specific temperature. A certain concentration of aluminum oxide solution was prepared and applied to a polishing plate. The sample was polished until the surface was smooth. The sample was then rinsed with deionized water, cleaned with anhydrous ethanol, and air-dried to complete the polishing process. Next, a small amount of silver paste was carefully applied evenly to one surface of the sample and then placed in an oven to dry. The same process was applied to the other surface, and once drying was complete, the sample was allowed to cool to room temperature. Finally, the edges of the silver paste on the sample were lightly polished with fine sandpaper to prevent electrical short-circuiting between the two silver-coated electrode surfaces. After polishing, the sample was ready for use. The polarization conditions used in this paper were as follows: polarization field strength of 3 kV/mm, polarization temperature of 120 °C, and polarization time of 30 min.

#### 2.2.3. Preparation Method of Three-Phase Composite Materials

The 0.6BaTiO_3_/0.4PVDF composite materials with relatively high piezoelectric performance and good mechanical deformation characteristics were selected as the matrix. The specific process was as follows.

1. Weighing: The pre-baked BaTiO_3_ ceramic, the dried PVDF polymer, and the BP2000 conductive carbon black, or the m5 graphene, were weighed according to a specific ratio.
2. Mixing: The conductive particles and polymer were placed in a beaker. Anhydrous ethanol was added (with the liquid level higher than the solvent by 2 cm), and the beaker was placed in an ultrasonic cleaner for ultrasonic agitation until a uniform mixture was obtained. This process took approximately 30 min.
3. Three-phase mixing of BaTiO_3_/PVDF/conductive particles: The PVDF/conductive particle suspension and BaTiO_3_ ceramic particles were placed in a ball mill jar and ground for about 40 min. The resulting three-phase liquid was then placed in an oven at 80 °C to dry.
4. Hot Press Forming: The dried three-phase mixture was placed in a mortar and ground into powder. The mixed powder was then added to a hot press mold for hot pressing, with the following conditions: 25 MPa, 180 °C, 30 min.
5. High-Pressure Polarization: After polishing, the sample, with a diameter of about 13 mm and thickness of about 1 mm, was placed in an oil bath for polarization under the following conditions: 3 kV/mm, 120 °C, 30 min. After polarization, the samples were dried and discharged for 24 h before testing.

### 2.3. Electrical Performance of Composite Materials

#### 2.3.1. Dielectric Properties

The magnitude of the dielectric constant indicated the degree of polarization and the ability of the piezoelectric element to store electrical charge. Since the capacitance of piezoelectric materials depended on the test frequency, the dielectric constant also changed with frequency. The relative dielectric constant was the ratio of the dielectric constant to the vacuum dielectric constant and was commonly used to measure the dielectric properties of piezoelectric materials. The electrical conductivity, dielectric constant ε, and relative dielectric constant ε_r_ were measured using an LCR meter (HIOKI ELECTRIC CO., Ueda, Japan, IM-3536). The frequency test range was 1000 Hz ~ 1 MHz.

The magnitude of dielectric loss reflected the degree of bonding between ceramic particles and the polymer matrix. The dielectric loss was characterized by the tanδ of the loss angle δ, which was measured using an LCR meter (HIOKI ELECTRIC CO., Ueda, Japan, IM-3536). The test was repeated three times and the average value was taken as the test data.

#### 2.3.2. Piezoelectric Properties

The piezoelectric strain constant reflected the linear response coefficient between mechanical quantities (stress or strain) and electrical quantities. When the direction of applied stress was aligned with the polarization direction of the piezoelectric ceramic, strain occurred on the surface of the ceramic, generating an electrical charge. The piezoelectric strain constant in the test was measured by the quasi-static d_33_ tester of model ZJ-3AN produced by the Institute of Acoustics, Chinese Academy of Sciences (Beijing, China). The test was repeated three times and the average value was taken as the test data.

Based on the positive piezoelectric effect, piezoelectric materials generate electrical displacement when subjected to external load, and this property is represented by the piezoelectric voltage constant *g*_33_, measured in V×m/N. It is often expressed as the ratio of the piezoelectric strain constant *d*_33_ to the dielectric constant ε, and it can be calculated by Equation (1).

(1) $$g_{33}=\frac{d_{33}}{ℇ}=\frac{d_{33}}{{ℇ}_{r}{ℇ}_{0}}$$

where *d*_33_ is the piezoelectric strain constant; ℇ*_r_* is the relative dielectric constant; ℇ_0_ is the vacuum dielectric constant, ℇ_0_ = 8.85 × 10^−12^ F/m.

### 2.4. Mechanical Performance of Composite Materials

Uniaxial compression tests were conducted on the samples using an electronic universal testing machine (MTS Industrial Systems (China) Co., Ltd., Shenzhen, China, CMT5105) to determine the compressive strength of piezoelectric composite materials. The samples were disk-shaped piezoelectric composite materials (13 mm diameter, 1 mm thickness). The loading rate was 0.1 mm/min., with 3 samples for each test group; we performed parallel tests and took the arithmetic mean of the results from the three samples as the measured value. The compressive strength of the piezoelectric composite materials was calculated using Equation (2).

(2) $$f_{cu}=\frac{F}{A}$$

where *f_cu_* is the compressive strength of the sample (MPa); *F* is the ultimate load (N); *A* is the compressed area (mm^2^).

### 2.5. Microstructure Characterization of Composite Materials

The microstructural morphology of the composite material surface was observed using secondary electron diffraction imaging with the S-570 model Environmental Scanning Electron Microscope (SEM) from Hitachi, Marunouchi, Japan.

## 3. Results and Discussion

### 3.1. The Electrical Performance of BaTiO_3_/PVDF Composite Materials

**Figure 3:**
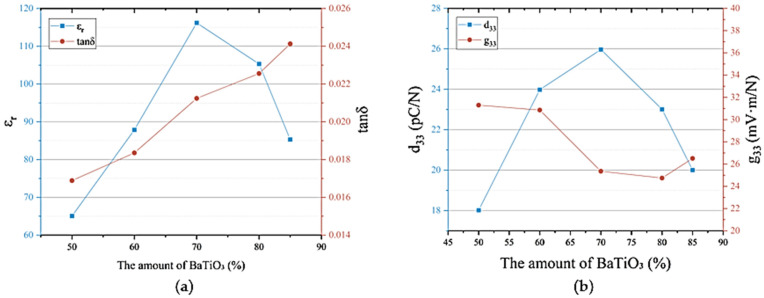
The electrical performance of BaTiO_3_/PVDF composite materials: (**a**) dielectric properties; (**b**) piezoelectric properties.

Figure 3a demonstrates the variation in the dielectric properties of BaTiO_3_/PVDF composite materials concerning the volume fraction of BaTiO_3_. With the increase in the BaTiO_3_ volume fraction in the composite materials, the ε_r_ value of BaTiO_3_/PVDF composite materials showed a trend of rapid increase followed by a sharp decrease. When the volume fraction of BaTiO_3_ increased from 50% to 70%, the ε_r_ value of the composite materials increased from 65 to 116, with an increase of more than 78%. When the volume fraction of BaTiO_3_ increased from 70% to 85%, the ε_r_ value of the composite materials decreased from 116 to 85, with a decrease of about 27%. With the increase in the BaTiO_3_ volume fraction in BaTiO_3_/PVDF composite materials, the tanδ value showed a gradual increase, and the tanδ value of the composite materials increased from 0.017 to 0.023 when the volume fraction of BaTiO_3_ increased from 50% to 85%, which was a smaller increase.

Figure 3b shows the relationship curve of the piezoelectric properties of BaTiO_3_/PVDF composite materials with the BaTiO_3_ volume fraction. With the gradual increase in the BaTiO_3_ volume fraction, the d_33_ value of the composite materials showed a gradual increase first and then a decreasing trend. When the volume fraction of BaTiO_3_ increased from 50% to 70%, the d_33_ value of the composite materials increased from 18 pC/N to 26 pC/N, which was an increase of nearly 39%. When the BaTiO_3_ volume fraction exceeded 70%, a sharp decrease in the d_33_ value occurred, and the d_33_ value decreased to 19 pC/N when the BaTiO_3_ volume fraction was 85%. The g_33_ value showed a decreasing and then increasing trend with the BaTiO_3_ volume fraction, but the overall trend was more stable. This was because the g_33_ value was jointly determined by the d_33_ value and the ε value. When the volume fraction of BaTiO_3_ was less than 70%, the increase rate of the d_33_ value of the composite was smaller than the increase rate of the ε value, so the decrease in the g_33_ value was obvious. When the volume fraction of BaTiO_3_ was between 70% and 80%, the decrease rate of the d_33_ value of the composite was comparable to the decrease rate of the ε value, so the decrease in the g_33_ value was small, at only 0.6 mV × m/N. When the BaTiO_3_ volume fraction was greater than 80%, the decrease rate of the d_33_ value of the composites was less than the decrease rate of the ε value, and the g_33_ value showed a weak increasing trend.

This was because, in BaTiO_3_/PVDF composite materials, with the increase in BaTiO_3_ content, it gradually occupied the dominant position of the composite materials. The increase in ceramic-phase content could improve the dielectric properties of the composite materials. With the increase in ceramic-phase content, the agglomeration phenomenon of BaTiO_3_ appeared, the interfacial defects and voids of the ceramic phase and the polymer matrix increased, and the symmetry of the crystal lattice was seriously disturbed by insufficient polarization. With the increase in the content of the ceramic phase, the polymer-based content decreased accordingly, which caused the polymer wrapping the ceramic particles to have a serious lack of ceramic structure due to loosening; the compatibility of the interface between the two phases deteriorated, and a serious thermal breakdown even occurred. Therefore, the content of the BaTiO_3_ volume fraction should not be more than 70%, and the composite material’s relative dielectric constant should be adjusted to obtain the maximum value. An optimum value of the ceramic-phase volume fraction (70%) existed in BaTiO_3_/PVDF composites and a maximum d_33_ value (26 pC/N) also existed at this optimum value.

### 3.2. The Electrical Performance of Three-Phase Composite Materials Based on Electrical Conductivity

**Figure 4:**
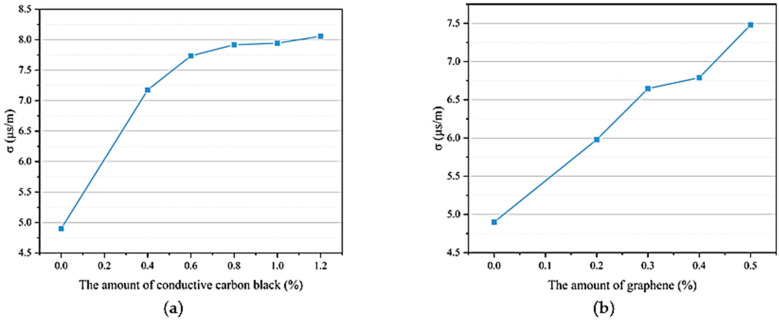
The influence of conductive particles on the electrical conductivity of three-phase composite materials: (**a**) conductive carbon black; (**b**) graphene.

Figure 4a shows the relationship between the electrical conductivity (*σ*) and the amount of conductive carbon black in three-phase composite materials at room temperature. The experiment measured the electrical conductivity of BaTiO₃/PVDF/conductive carbon black composite materials with mass ratios (conductive carbon black and PVDF) of 0.0%, 0.4%, 0.6%, 0.8%, 1.0%, and 1.2%. Figure 4b shows the relationship between the electrical conductivity of three-phase composite materials and the amount of graphene added at room temperature. The experiment measured the electrical conductivity of BaTiO₃/PVDF/graphene composite materials with mass ratios (graphene to BaTiO₃/PVDF composite material) of 0.0%, 0.2%, 0.3%, 0.4%, and 0.5%. The BaTiO₃/PVDF composite material itself had a certain degree of conductivity, approximately 4.9 μS/m. As conductive carbon black or graphene particles were added, the conductive pathways within the composite materials began to increase, and the conductivity showed an upward trend.

When the content of conductive carbon black or graphene was zero, in the composite materials, there were very few conductive pathways, and the significant difference in dielectric properties between the ceramic and the polymer resulted in a small electric field strength applied to BaTiO₃. This led to a relatively weak overall conductivity of the composite materials. When the content of conductive carbon black or graphene was low, the particles could only be dispersed individually within the composite materials and could not connect with each other, resulting in a limited contribution to the conductive pathways. As the conductive carbon black or graphene content increased, particles that could not previously connect began to form certain pathways. Additionally, the conductive carbon black and graphene particles shortened the movement distance of charge carriers during polarization, increasing the number and distribution of charge carriers in the composite materials, thereby significantly improving the overall conductivity of the composite materials. Moreover, the more conductive carbon black or graphene was added, the more pronounced this enhancement effect became. However, this increasing trend would not continue indefinitely because when the number of conductive particles in the composite materials reached a certain limit, the composite materials would be unable to polarize due to excessive leakage current during polarization, and might even experience severe breakdown. This was due to the significant agglomeration of conductive carbon black or graphene particles, forming larger conductive channels that led to a sharp increase in leakage current, causing the specimen to approximate a conductor and lose its ability to polarize.

Therefore, in the experiment, there was a maximum allowable amount of conductive carbon black, which was 1.2%. If the amount exceeded this value, the piezoelectric composite material would be broken down by high-voltage currents and could no longer be polarized. When the graphene content exceeded 0.5%, the specimen could no longer be polarized. Therefore, the maximum graphene content was determined to be 0.5%. At this point, the σ value was 7, approximately twice the initial value.

### 3.3. The Electrical Performance of Three-Phase Composite Materials Based on Dielectric Properties

#### 3.3.1. The Dielectric Properties of Three-Phase Composite Materials

**Figure 5:**
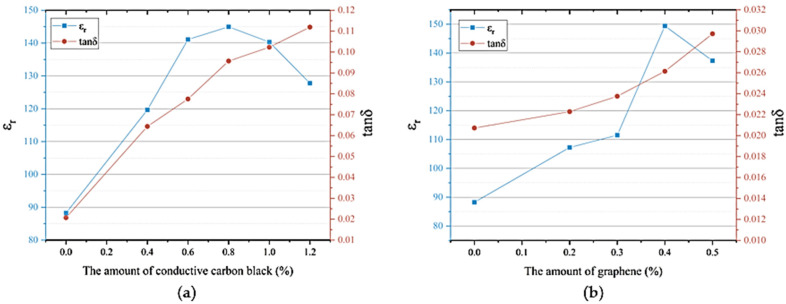
The influence of conductive particles on the dielectric properties of three-phase composite materials: (**a**) conductive carbon black; (**b**) graphene.

Figure 5a shows the relationship between the dielectric properties (relative dielectric constant ε_r_, dielectric loss tanδ) of BaTiO₃/PVDF/conductive carbon black composite materials and the amount of conductive carbon black. Figure 5b shows the relationship between the dielectric properties of BaTiO₃/PVDF/graphene composite materials and the amount of graphene added. The addition of conductive carbon black or graphene played a significant role in enhancing the relative dielectric constant of the composite material, but this increase in ε_r_ was accompanied by a rise in dielectric loss. The relative dielectric constant of piezoelectric composite materials reflected the efficiency of polarization and the amount of energy stored in the material. When no conductive carbon black or graphene particles were added, the ε_r_ value of the composite material was relatively low, only 88. When the carbon black content reached 0.4%, the relative dielectric constant increased from 88 to 119, an increase of about 39%. When the conductive carbon black content reached 0.8%, the relative dielectric constant peaked at 145. When the graphene content reached 0.2%, the ε_r_ value of the composite material reached 107, an increase of about 28%. When the graphene content was 0.4%, this enhancement reached its peak, causing the ε_r_ value of the BaTiO₃/PVDF/graphene composite material to reach its maximum value of 149. However, if the content of conductive materials was further increased, the ε_r_ value of the composite material decreased.

When the number of conductive carbon black or graphene particles was small, conductive particles could not form continuous conductive pathways, so they had minimal effect on polarization, and consequently, their contribution to increasing the relative dielectric constant was limited. However, as the number of conductive carbon black or graphene particles increased, the conductive particles were brought closer together, increasing the charge transfer channels. This also enhanced the electric field distribution in the ceramic phase, improving polarization efficiency and leading to an increase in the relative dielectric constant. When the content exceeded a certain threshold, excessive conductive carbon black or graphene particles agglomerated, increasing the overall leakage current, reducing the polarization efficiency, and correspondingly decreasing ε_r_. Once the leakage current exceeded a certain limit, the composite material could no longer polarize and even underwent breakdown.

Figure 5a and b also show the relationship between the tanδ value of the composite material and the amount of conductive carbon black or graphene added. It could be observed that the tanδ value increased gradually with the increasing content of conductive particles. This was because dielectric materials contained charge carriers that could conduct electricity. Under the influence of an external electric field, these charge carriers moved directionally, consuming a certain amount of electrical energy, which was then dissipated in the form of heat. When the amount of conductive carbon black or graphene was low, the movement of these charge carriers was weak, leading to a small dielectric loss that increased slowly. As the content of conductive particles increased, the charge carrier movement became more intense, and the dielectric loss gradually increased until the leakage current became large enough to prevent polarization. However, when the carbon black content was 0.8%, the tanδ value remained below 0.1, and at the maximum graphene content, the tanδ value remained below 0.03, indicating that the dielectric loss was still within a relatively low range.

#### 3.3.2. The Frequency-Dependent Dielectric Properties of Three-Phase Composite Materials

**Figure 6:**
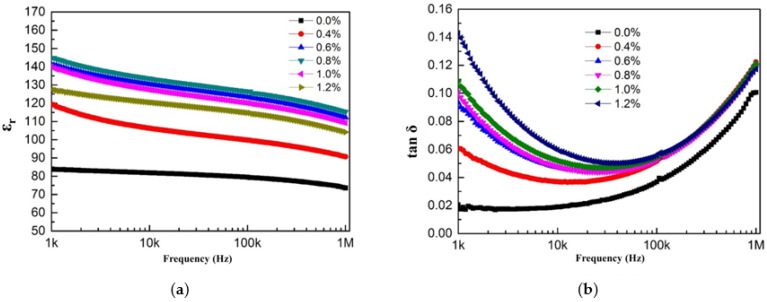
The frequency-dependent dielectric properties of BaTiO_3_/PVDF/conductive carbon black composite materials: (**a**) relative dielectric constant; (**b**) dielectric loss.

Figure 6 shows the relationship between the dielectric properties of BaTiO₃/PVDF/conductive carbon black composite materials and test frequency. As shown in Figure 6a, the ε_r_ value of BaTiO₃/PVDF/conductive carbon black composite materials gradually decreased with increasing test frequency. In composites with highly conductive material, the decrease rate of ε_r_ was faster in the low-frequency test range (*f* < 10⁴ Hz) than in the high-frequency test range (*f* > 10⁴ Hz). Figure 6b shows the variation in dielectric loss (tanδ) with test frequency for BaTiO₃/PVDF/conductive carbon black composite materials. As the test frequency increased, the tanδ initially decreased and then increased. In the low-frequency range (*f* < 10⁴ Hz), tanδ decreased as the test frequency increased. However, in the high-frequency range (*f* > 10⁴ Hz), tanδ increased with frequency, and the increase became more pronounced.

**Figure 7:**
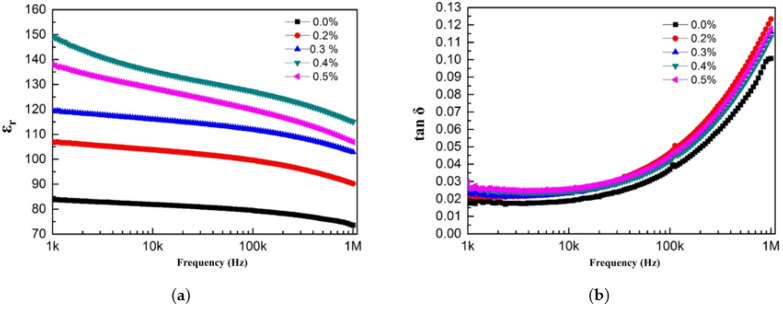
The frequency-dependent dielectric properties of BaTiO_3_/PVDF/graphene composite materials: (**a**) relative dielectric constant; (**b**) dielectric loss.

Figure 7 shows the relationship between the dielectric properties of BaTiO₃/PVDF/graphene composite materials and test frequency. As shown in Figure 7a, the ε_r_ value of BaTiO₃/PVDF/graphene composite materials gradually decreased with increasing test frequency. The same pattern of change as BaTiO₃/PVDF/conductive carbon black composite materials appeared. Figure 7b shows the relationship between the tanδ and test frequency for BaTiO₃/PVDF/graphene composite materials. As the test frequency increased, the tanδ gradually increased. In the low-frequency range (*f* < 10⁴ Hz), tanδ remained relatively stable with increasing frequency, indicating good low-frequency stability of the composite material. However, in the high-frequency range (*f* > 10⁴ Hz), tanδ increased sharply with frequency, and the increase became more pronounced.

This was mainly due to the interfacial polarization effect, as some charges accumulated and migrated between the polymer matrix and the barium titanate ceramic particles. The accumulation and migration of these charge carriers resulted in high-performance polarization, leading to a high dielectric constant. At lower frequencies, the charge carriers moved relatively slowly, and at the interface, they mainly accumulated. However, as the test frequency increased, the charge carrier migration accelerated sharply, generating more heat. This led to higher dielectric loss at higher frequencies, and the higher the test frequency, the larger and faster the increase in loss. In practical applications, the composite material should be limited to low-frequency environments to avoid severe breakdown phenomena.

### 3.4. The Electrical Performance of Three-Phase Composite Materials Based on Piezoelectric Properties

**Figure 8:**
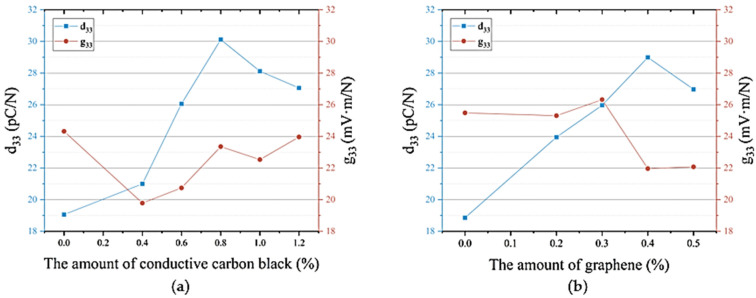
The influence of conductive particles on the piezoelectric properties of three-phase composite materials: (**a**) conductive carbon black; (**b**) graphene.

Figure 8a shows the relationship between the piezoelectric properties of BaTiO_3_/PVDF/conductive carbon black composite materials and the amount of conductive carbon black added. With the increase in the amount of conductive carbon black, the d_33_ value of the composite materials gradually increased. When the amount of conductive carbon black was less than 0.4%, the increase in d_33_ was slow, rising from 19 pC/N to 21 pC/N. When the carbon black content reached 0.8%, the d_33_ value increased from 21 pC/N to 30 pC/N, which represented an approximately 43% increase. When the carbon black content reached 0.8%, the enhancement effect peaked, resulting in the maximum piezoelectric performance of the composite material at this carbon black concentration. Figure 8a also shows the relationship between the g_33_ value of BaTiO_3_/PVDF/conductive carbon black composite materials and the amount of conductive carbon black added. Since the g_33_ value was determined by both the piezoelectric strain constant and dielectric constant (g_33_ = d_33_/ε), as the amount of conductive carbon black increased when the increase in d_33_ was greater than the increase in ε, the g_33_ value tended to increase. Conversely, when the increase in d_33_ was smaller than that in ε, the g_33_ value tended to decrease. Therefore, the relationship was not entirely regular. It could be seen that the piezoelectric voltage constant reached a large value of 23 mV × m/N near the optimal conductive carbon black content.

Figure 8b shows the relationship between the piezoelectric properties of BaTiO_3_/PVDF/graphene composite materials and the amount of graphene added. With the increase in graphene content, the d_33_ value of the composite material first increased and then decreased. In the absence of graphene, the BaTiO_3_/PVDF composite material had a low piezoelectric performance, with a d_33_ value of only 19 pC/N. At a low graphene content of 0.2%, the d_33_ value increased to 24 pC/N, representing a 21% increase. At a graphene content of 0.4%, the d_33_ value reached its maximum of 29 pC/N, more than 1.5 times the initial value. When the graphene content exceeded 0.4%, the piezoelectric properties declined. If the graphene content exceeded 0.5%, the BaTiO_3_/PVDF composite material could not be polarized due to excessive leakage current. In conclusion, the maximum graphene content was 0.5%, and the optimal content was 0.4%, where the composite material achieved the best d_33_ value. Figure 8b also shows the trend of g_33_ values for BaTiO_3_/PVDF/graphene composite materials with varying amounts of conductive carbon black. The g_33_ values for graphene content less than 0.3% were higher, all above 25 mV × m/N. When the graphene content exceeded 0.3%, the g_33_ value decreased to around 22 mV × m/N.

With the addition of conductive particles, more conductive pathways were formed within the polymer matrix. On the one hand, conductive carbon black or graphene itself had a higher dielectric constant, which reduced the dielectric constant difference between the ceramic phase and the polymer matrix. This increased the distribution of the polarization field in the ceramic phase, improving polarization efficiency and promoting an increase in d_33_. On the other hand, the addition of conductive carbon black or graphene reduced the distance between charge carriers, creating more charge transport pathways, which enhanced the overall polarization efficiency of the composite material and improved its piezoelectric properties. However, with the continued increase in conductive carbon black or graphene particles, agglomeration could even occur. This agglomeration increased the internal conductivity of the composite material, causing the material to approach a conductor. The leakage current during polarization increased sharply, leading to polarization failure and even severe breakdown.

### 3.5. The Mechanical Performance of Three-Phase Composite Materials

**Figure 9:**
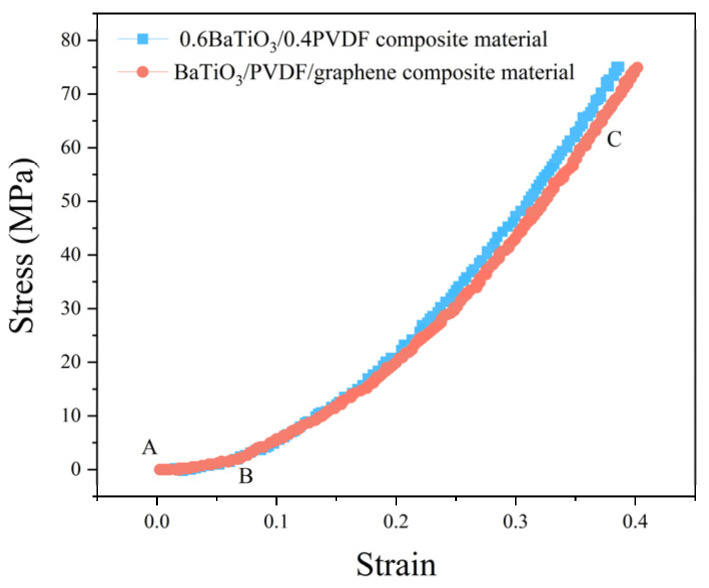
The influence of conductive particles on the mechanical performance of three-phase composite materials.

The addition of conductive particles had a significant impact on improving the mechanical properties of composite materials. Since the particle sizes of conductive carbon black and graphene were relatively small, and their contents were negligible compared to the ceramic phase and polymer matrix, the results indicated that the different content levels of conductive particles had a minimal effect on the mechanical properties of the composite materials. The strain–stress relationship curves were almost identical. Therefore, the 0.6BaTiO_3_/0.4PVDF composite material was set as the control group. BaTiO_3_/PVDF/graphene composite material and 0.6BaTiO_3_/0.4PVDF composite material were compared. Figure 9 shows the strain–stress relationship curve. In the AB segment, the graphene-added composite material gradually started to produce compressive deformation under load, and the stress started to increase slightly with increasing strain. In this stage, the composite material specimen underwent nonlinear deformation under mechanical load, and the deformation trend was similar to that of the composite material without conductive particles, with only minor differences in the deformation amount. In the BC segment, the composite material specimen also exhibited a linear deformation curve, displaying elastic body characteristics. At low strain, it was only the PVDF that was compressed. When the strain was increased, the BaTiO_3_ grains were compressed together, so that in the high-strain limit, the high modulus of the ceramic took over. After compression, the specimen became more compact and thinner, with no noticeable brittle fracture or electrical loss. This suggested that the material would not fail under vehicular loads, and its road performance met the compressive strength requirements.

### 3.6. The Microstructure Characterization of Three-Phase Composite Materials

**Figure 10:**
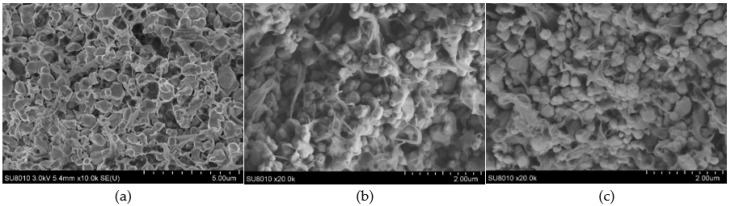
The microscopic morphology of three-phase composite materials: (**a**) BaTiO_3_/PVDF composite materials; (**b**) 1.2% conductive carbon black in BaTiO_3_/PVDF composite materials; (**c**) 0.5% graphene in BaTiO_3_/PVDF composite materials.

Figure 10 shows the microstructure of BaTiO₃/PVDF composite materials with 1.2% conductive carbon black and 0.5% graphene. Since the conductive materials had little effect on the microstructure of the composites, only images at their respective maxima are shown. It could be observed that the PVDF polymer matrix formed a flocculent structure that completely wrapped the ceramic particles, creating a stacked overall structure. The ceramic particles were uniformly dispersed within the polymer. From the cross-section, it was evident that the interface between the ceramic phase and the polymer matrix was compatible, with no obvious large gaps or dielectric defects at the interface. The overall structure of the composite material was compact and complete. Due to the low amount of carbon black particles and their small size, the presence of conductive carbon black or graphene particles was not visible. The addition of conductive carbon black and graphene particles had no significant impact on the structure of the composite material.

## 4. Conclusions

In this study, based on the effective electric field theory, the dielectric constant of the polymer matrix was enhanced to reduce the dielectric constant gap between the polymer matrix and the ceramic phase, thereby improving the polarization efficiency by increasing the electric field strength applied to the piezoelectric ceramic functional phase. Conductive carbon black and graphene, which have superior conductivity, were used as modification materials. Using the traditional hot-pressing molding process, BaTiO_3_/PVDF/conductive carbon black composite materials with mass ratios (carbon black to PVDF mass ratio) of 0.4%, 0.6%, 0.8%, 1.0%, and 1.2%, and BaTiO_3_/PVDF/graphene composite materials with mass ratios (graphene to BaTiO_3_/PVDF composite material mass ratio) of 0.2%, 0.3%, 0.4%, and 0.5% were prepared. The electrical performance was studied by the conductivity, dielectric properties, and piezoelectric properties of the three-phase composite materials. Then, the mechanical properties of the composite material were investigated by load compression tests. Finally, the microstructure of the composite materials was studied. The following research conclusions were drawn:

1. In BaTiO_3_/PVDF composite materials, the addition of conductive carbon black and graphene improved the electrical conductivity. An increase in the BaTiO_3_ content failed to improve the overall electrical properties. The addition of conductive carbon black and graphene in the composite materials would better improve the polarization efficiency. Both BaTiO_3_/PVDF/conductive carbon black and BaTiO_3_/PVDF/graphene composite materials exhibited good frequency stability. As the amount of conductive particles increased, this enhancement became more pronounced. However, further addition of conductive particles led to a decline in the dielectric properties, eventually interrupting the polarization process due to excessive leakage current and even causing severe breakdown. In the BaTiO_3_/PVDF/conductive carbon black composite material, the optimal amount of carbon black was 0.8%. The maximum addition amount was 1.2%. In the BaTiO_3_/PVDF/graphene composite material, the optimal amount of graphene was 0.4%. The maximum addition amount was 0.5%, and beyond this, polarization could not occur.
2. The addition of conductive particles in BaTiO_3_/PVDF composite materials significantly improved the piezoelectric properties of the composite materials. This effect initially increased and then decreased with the amount of conductive particles added, indicating the presence of an optimal addition level for the conductive particles. Specifically, in the BaTiO_3_/PVDF/conductive carbon black composite materials, the optimal amount of carbon black was 0.8%, where the d_33_ value increased from 19 pC/N to 30 pC/N, an improvement of about 60%. In the BaTiO_3_/PVDF/graphene composite materials, the optimal amount of graphene was 0.4%, where the d_33_ value increased from 19 pC/N to 29 pC/N, an improvement of about 53%.
3. The addition of conductive particles had a minimal effect on the mechanical properties of composite materials. The three-phase added composite material met road use requirements. The overall structure of the composite materials was compact, with a clear wrapping effect of the polymer, and good interface compatibility. The addition of conductive carbon black and graphene had no significant impact on the structure of the composite materials. Therefore, the composite piezoelectric materials prepared with lead-free BaTiO_3_ piezoelectric ceramics, polymer piezoelectric materials PVDF, and conductive particles had excellent performance.

## Data Availability

Dataset available on request from the authors.

## Abbreviations

The following abbreviations are used in this manuscript:

PZT	Titanate
BaTiO_3_	Barium titanate
PVDF	Polyvinylidene fluoride
